# Novel On-Demand 3-Dimensional (3-D) Printed Tablets Using Fill Density as an Effective Release-Controlling Tool

**DOI:** 10.3390/polym12091872

**Published:** 2020-08-20

**Authors:** Rishi Thakkar, Amit Raviraj Pillai, Jiaxiang Zhang, Yu Zhang, Vineet Kulkarni, Mohammed Maniruzzaman

**Affiliations:** Pharmaceutical Engineering and 3D Printing (PharmE3D) Labs, Division of Molecular Pharmaceutics and Drug Delivery, College of Pharmacy, The University of Texas at Austin, Austin, TX 78705, USA; rishithakkar@utexas.edu (R.T.); arpillai@utexas.edu (A.R.P.); Jiaxiang.Zhang@austin.utexas.edu (J.Z.); yu.zhang@utexas.edu (Y.Z.); vineetkulkarni@utexas.edu (V.K.)

**Keywords:** fused deposition modeling, additive manufacturing, HPMC-AS, controlled release, personalized medication, hot-melt extrusion, amorphous solid dispersions

## Abstract

This research demonstrates the use of fill density as an effective tool for controlling the drug release without changing the formulation composition. The merger of hot-melt extrusion (HME) with fused deposition modeling (FDM)-based 3-dimensional (3-D) printing processes over the last decade has directed pharmaceutical research towards the possibility of printing personalized medication. One key aspect of printing patient-specific dosage forms is controlling the release dynamics based on the patient’s needs. The purpose of this research was to understand the impact of fill density and interrelate it with the release of a poorly water-soluble, weakly acidic, active pharmaceutical ingredient (API) from a hydroxypropyl methylcellulose acetate succinate (HPMC-AS) matrix, both mathematically and experimentally. Amorphous solid dispersions (ASDs) of ibuprofen with three grades of AquaSolve^TM^ HPMC-AS (HG, MG, and LG) were developed using an HME process and evaluated using solid-state characterization techniques. Differential scanning calorimetry (DSC), powder X-ray diffraction (pXRD), and polarized light microscopy (PLM) confirmed the amorphous state of the drug in both polymeric filaments and 3D printed tablets. The suitability of the manufactured filaments for FDM processes was investigated using texture analysis (TA) which showed robust mechanical properties of the developed filament compositions. Using FDM, tablets with different fill densities (20–80%) and identical dimensions were printed for each polymer. In vitro pH shift dissolution studies revealed that the fill density has a significant impact (F(11, 24) = 15,271.147, *p* < 0.0001) and a strong negative correlation (r > −0.99; *p* < 0.0001) with the release performance, where 20% infill demonstrated the fastest and most complete release, whereas 80% infill depicted a more controlled release. The results obtained from this research can be used to develop a robust formulation strategy to control the drug release from 3D printed dosage forms as a function of fill density.

## 1. Introduction

The development and advancements in 3-dimensional (3-D) printing over the last decade have directed pharmaceutical research towards the possibility of printing personalized or patient-specific drug delivery systems [1,2]. These dosage forms have the potential to tailor therapies with the most efficient, as well as effective response and safety margins [3]. In recent times, researchers have been exploring the applications of different 3-D printing platforms for producing pharmaceutical dosage forms [4] such as selective laser sintering (SLS) [5,6,7], stereolithographic 3-D printing (SLA) [8], binder jet printing [9], and fused deposition modeling (FDM) based 3-D printing [10]. The current literature lacks insights on the impact of processing parameters such as fill density on the performance of these dosage forms which we have attempted to explore as a part of this research study.

Extensive research has been conducted on the application of using SLS in the printing of medicine [11], selected works include printing of immediate-release [5], sustained/controlled/modified release [12,13], multi-drug containing [14], and orally disintegrating dosage forms [15]. Although SLS has proven to be a versatile solvent-free technique for printing dosage forms, most of the drug candidates used in the conducted research belong to the class I of the biopharmaceutical classification system (BCS). Amongst the aforementioned platforms, only FDM allows the use of hot-melt extrusion (HME) based filaments which are known for their capabilities of solubility enhancement, specifically for BCS class II drugs [16,17], which makes it a preferred platform for drugs with poor water solubility over the other techniques. Moreover, FDM is known to have the most versatility in terms of designing 3-D structures [18,19,20] and the available pharmaceutical polymers compatible with the process [20,21,22,23]. It has been recognized that FDM has the most immediate potential for unit dose fabrication as it uses polymeric filaments which could be customized or tuned to the specific needs of the printed products. These filaments with homogenous drug-polymer blends can be optimized employing HME processing where drug disperses into thermoplastic polymeric matrices. In recent years, FDM has been used to produce immediate release [24], sustained-release [25], gastro retentive [26,27], and controlled release [28] drug delivery systems, in the form of tablets [29], as well as caplets [25] with one or more than one active pharmaceutical ingredients (API) [30].

Infill density (also known as %infill, or fill density) by definition is a print parameter which controls the percent of the printed region within the walls, and the top- and bottom layers of the design. It can be controlled during the slicing step of the printing process. Even though ample research on designing dosage forms with novel pharmaceutical applications is being conducted, not much research has been dedicated to inter-relate print parameters and their impact on the performance of the FDM-printed devices. Goyanes, A., and colleagues (2015) have discussed the development of modified-release formulations of two aminosalicylate isomers used in the treatment of inflammatory bowel disease (IBD) i.e., 5-aminosalicylic acid (5-ASA, mesalazine) and 4-aminosalicylic acid (4-ASA). The study used a commercially manufactured PVA filament, where the drug was absorbed on to the said filament. The study tested the performance of the FDM printed tablets at different fill densities (10%, 50%, and 90%) as a secondary objective of the study and based on the observations the study concluded that the 10% infill tablets observed a complete release after 4 h dissolution, but both the 50% and 90% infill tablets showed burst release followed by a slow-release. G. Verstraete and colleagues (2018) conducted a similar study to explore the sustained release applications of FDM for high drug-loaded (>30%, *w*/*w*), thermoplastic polyurethane (TPU)-based dosage forms. As a secondary objective, tablets with different fill densities (25%, 50%, 75%, and 100%) were compared and observation-based conclusions were drawn where the observed release profiles were attributed to several factors including the type of the polymer used, the drug type, and the porosity of tablets. Similar observations can be drawn from prior publications from Yang and colleagues (2018) and Goyanes and colleagues (2017) which have used ethyl cellulose and HPMC-AS based matrices, respectively. In nutshell, numerous publications have briefly observed the impact of fill density but have not confirmed its significance and correlation with the performance of the tablets using statistical tools, which is understandable as it was never the driving objective of these studies. Thereby, they merely provide an inkling of the association between the dependent (performance) and independent (fill density) variables. Moreover, the prior studies conducted so far use different filament manufacturing/loading platforms, different polymer bases (each with its distinct release mechanism), and different drug classes (with different physicochemical properties). This highlights the importance and the need for studies aiming to determine the effect of processing parameters such as fill density on each pharmaceutically acceptable polymer and drug class.

This paper discusses the impact of fill density on the in vitro performance of the tablets. To make this research comprehensive, a poorly water-soluble, weakly acidic model drug, ibuprofen (IBU) was loaded in three different grades of the hydroxypropyl methylcellulose acetate succinate (HPMC-AS) polymers i.e., HG, LG, and MG using hot-melt extrusion processing. The key difference between the grades is the amount of acetyl (LG < MG < HG) and succinoyl (HG < MG < LG) substitution which correlates with the polymer’s aqueous pH-dependent solubility. The manufactured filaments (diameter ~2.5 mm) were characterized using modulated differential scanning calorimetry (mDSC), powder X-ray diffraction analysis (pXRD), polarized light microscopy (PLM), and Fourier transform–infrared spectroscopy (FT–IR) to confirm the formation of amorphous solid dispersions (ASDs). The compatibility of the manufactured filaments with the FDM printer was evaluated as a function of mechanical profiles using texture analysis by comparing the filaments with polylactic acid (PLA) and polyvinyl alcohol (PVA)-based reference material [21]. Each of the drug-loaded polymeric filament was used for 3-D printing of tablets with different fill densities, where the rest of the print parameters were kept constant. These printed tablets were evaluated for their in vitro performance using a pH shift dissolution protocol as the polymers under investigation have a pH-dependent solubility. Further, the dissolution profiles were compared using appropriate statistical tests and kinetic models. It was observed that fill density had a significant impact on the release kinetics of the drug from the polymer, where the tablets with the least infill depicted the fastest drug release and the ones with a higher infill had a more sustained release.

Such a correlation can have potential applications in the printing of personalized medication, where the release patterns of the dosage forms can be controlled by manipulating the infill of the tablets as per the patient’s needs and the properties of the API without changing the filament components/composition or the design of the tablets. Moreover, this study provides deeper insights into the impact of print parameters on the performance of 3-D printed dosage forms which would help investigators gain a better understanding of the process variables.

## 2. Materials and Methods

### 2.1. Materials

2-(4-Isobutylphenyl) propionic acid (Tokyo Chemical Industry Co., Ltd., Tokyo, Japan, Lot no. I2HJDND), AquaSolve^TM^ HPMC-AS HG (Ashland Specialty Ingredients, Wilmington, DE, USA, Lot no. 65G-810004), AquaSolve^TM^ HPMC-AS MG (Ashland Specialty Ingredients, Wilmington, DE, USA, Lot no. 60G-810002), AquaSolve^TM^ HPMC-AS LG (Ashland Specialty Ingredients, Wilmington, DE, USA, Lot no. 55G-910001), Tough PLA filaments, natural filaments PVA (Ultimaker, Geldermalsen, The Netherlands) were used. All other chemicals, solvents, and reagents used in this study were of analytical grade and obtained from Fisher Scientific (Fair Lawn, NJ, USA).

### 2.2. Hot-Melt Extrusion Process

**Figure 1:**
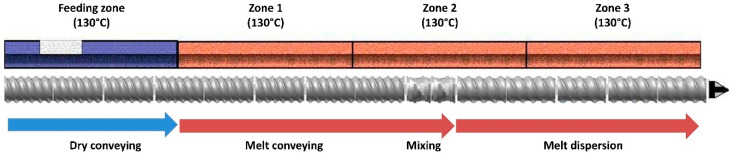
Screw design was selected for processing of the active pharmaceutical ingredient (API) and polymer physical blends.

Preliminary screening of polymers for ibuprofen was conducted using Hansen solubility parameters and thermal analysis of the API and the excipients (differential scanning calorimetry, the method is described in later sections). Based on the thermal investigation of the API and the polymers, processing parameters outlined in Table 1 were selected. Physical mixtures containing 20% (*w*/*w*) API and 80% (*w*/*w*) polymers (HPMC-AS HG, MG, and LG) were prepared using geometric dilution. These physical blends were introduced to the extruder using a calibrated volumetric feeder (Brabender twin screw feeder with stirring agitators, Brabender Technologie, Duisburg, Germany). The blend was processed using a co-rotating twin-screw extruder with 16 mm outer diameter (OD) (Nano-16 Twin screw extruder, Leistritz, Nuremberg, Germany) and an optimized screw configuration depicted in Figure 1. The molten mass was extruded through a 2.5 mm die and the diameter of the filaments was monitored constantly using a Vernier caliper. The collected filaments were stored in a validated desiccator for further use and characterization. A single batch consisted of 50 g of the physical mixture, three batches were prepared for each polymer.

### 2.3. Solid-State Characterization

#### 2.3.1. Thermal Investigation

Pure crystalline API, polymers, API-polymer physical mixtures (PM), and manufactured amorphous solid dispersions were subjected to mDSC (DSC Q20, TA^®^ instruments, New Castle, DE, USA) analysis to identify their solid state and important thermal events [31]. Approximately, 5–15 mg of the samples were weighed in standard DSC aluminum pans and sealed using standard aluminum lids (DSC consumables incorporated, Austin, MN, USA) using a calibrated balance. The samples were subjected to a temperature range from 35 °C to 175 °C, with a ramp rate of 3 °C/minute and modulation of 0.30 °C every 50 s. The data were collected, analyzed, and presented as a plot of temperature (°C) versus reverse heat flow (mW).

#### 2.3.2. Powder X-ray Diffraction Studies

The crystallinity of all the components, physical mixtures, extrudates, and the 3-D printed tablets of ibuprofen and HPMC-AS HG after in vitro dissolution studies were investigated using pXRD. The samples were prepared and loaded on to the magnetic sample cell. The cells were then placed in the sample holder of the benchtop pXRD instrument (MiniFlex, Rigaku Corporation, Tokyo, Japan). Finally, the samples were scanned from a 2θ angle of 5 to 60 degrees [32], with a scan speed of 2°/min. The scan step was maintained at 0.02 degrees, the resultant scan resolution was found to be 0.0025. The current and the voltage of the system were maintained at 15 mV and 45 V, respectively. Collected data were analyzed.

#### 2.3.3. Polarized Light Microscopy

The crystallinity of the API, the extrudates, and later on the 3-D printed tablets of ibuprofen and HPMC-AS HG after in vitro dissolution studies were assessed using PLM analysis. An Olympus BX53 polarizing photomicroscope (Olympus America Inc., Webster, TX, USA) equipped with Bertrand Lens was used for analyzing the samples. Briefly, the powdered sample was spread out evenly on a glass slide to avoid clumping. The excess powder was dusted off and the samples were covered using a coverslip. The slide was then placed onto the microscope stage and observed under a 10× magnification. Presence of birefringence, a property observed in crystalline substances was considered as an indication of the presence of crystallinity. Images were captured using QICAM Fast 1394 digital camera (QImaging, Surrey, BC, Canada) under light using a 530 nm compensator (U-TP530, Olympus^®^ corporation, Shinjuku City, Tokyo, Japan) and dark background conditions. Captured images were analyzed using Linksys 32 software^®^ (Linkam Scientific Instruments Ltd., Tadworth, UK).

#### 2.3.4. Fourier Transform–Infrared (FT–IR) Spectroscopic Analysis

The intermolecular interactions between the polymers (HPMC-AS HG, MG, and LG) and the drug were investigated using FT–IR analysis (iS50 FT–IR equipped with a SMART OMNI-Sampler, Nicolet, ThermoFisher Scientific, Waltham, MA, USA). Then, 20–25 mg of samples of pure drug, pure polymers, drug-polymer physical mixtures, and powdered extruded filaments were analyzed for % transmittance from 3100–700 cm^−1^, at a resolution of 4 cm^−1^ (64 scans per run), and background was collected before every run. The collected spectra were analyzed and assessed for weak intermolecular interaction on the OMNIC^TM^ series software (ThermoFisher Scientific, Waltham, MA, USA).

### 2.4. Texture Analysis

Flexibility and brittleness properties of the manufactured filaments were evaluated along with ‘Tough PLA’ and ‘Natural PVA’ filaments as the reference material to represent the printability of the filaments [33]. For flexibility and brittleness analysis, extruded filament samples from each batch were collected and cut into 30 mm in length. TA-XT2 analyzer (Texture Technologies Corp, New York, NY, USA) along with the TA-92N mini 3-point bend apparatus set having a 20 mm supporting gap was used to test the brittleness and flexibility of the extruded filaments. The moving speed of the blades was set to 10 mm/s and the target mode was set to distance, where the probe exerted force on the filament for the distance of 15 mm on contact. Each single formulation filaments were repeated 10 times. Breaking distance and load force/stress data were collected and analyzed by Exponent software (Stable Microsystems, Godalming, Surrey, UK).

### 2.5. Fused Deposition Modeling-Based 3-Dimensional Printing

The tablets were designed using the 3D builder software (Microsoft Corporation, Redmond, Washington, DC, USA). The designed tablets were then transferred to the slicing software (Ultimaker Cura, Utrecht, The Netherlands). The designed tablets were sliced with different infill densities (20%, 40%, 60%, and 80%). The rest of the processing parameters (Table 2) were kept constant to isolate the impact of the variable on tablet performance. The suitable filaments were loaded in the FDM 3-D printer (Ultimaker S3, Utrecht, The Netherlands) and tablets having the dimensions 10 × 10 × 4 mm were printed for each polymer and stored in a validated desiccator for future use and characterization. The morphology of the 3-D printed tablets was investigated using digital microscopy (Dino light, Torrance, CA, USA).

### 2.6. Dosage form Performance (In Vitro Drug Release Testing)

To test the performance of the tablets, a pH shift dissolution testing protocol was developed for the USP type II dissolution apparatus (Paddle type). The prepared tablets were first exposed to 750 mL of hydrochloric acid (HCl)-potassium chloride (KCl) buffer (pH 2, 0.1 M) for 2 h in a 900 mL dissolution vessel. After 2 h; 150 mL of phosphate buffer (pH 6.8, 0.1 M) was added to each vessel making up a final volume of 900 mL and shifting the pH from 2 to 6.8. The tablets were subjected to pH 6.8 for 6 h. The dissolution system (Vankel VK 7000, Agilent Technologies, Santa Clara, CA, USA) was operated at 37.5 °C, and 50 RPM. One milliliter of media was withdrawn and filtered (10 µm polyethylene dissolution filters, Fisher Scientific, Waltham, MA, USA) using an autosampler (Vankel VK 8000, Agilent Technologies, Santa Clara, CA, USA) at predetermined time points and the vessels were replaced with fresh buffer (HCl-KCl/Phosphate buffer). The collected samples were diluted two-fold with acetonitrile (HPLC grade) and the amount of API was estimated using the described method of analysis. To gain better clarity of the protocol please refer to the recipe table in the supplementary data (Table S1) The same protocol was followed for all manufactured tablets with different grades of the polymer and the dissolution studies were conducted in triplicates (*n* = 3). Three tablets for each sample were printed to have a uniform mass and low standard deviation amongst the group, this was done to limit variability due to additional other factors apart from fill density (Table S2). The morphology of the 3-D printed tablets after the dissolution studies were investigated using a digital microscope. The 3-D printed tablets were not compared with the crystalline drug as the solubility advantage of ibuprofen ASDs over its crystalline counterpart has been previously reported and demonstrated [34].

### 2.7. Method of Analysis

Estimation of ibuprofen was conducted using a reverse phase-high performance liquid chromatographic (RP-HPLC) analysis (Agilent 1100 series, Agilent Technologies, Santa Clara, CA, USA) with a 25 cm × 4.6 mm, 5 µm particle size, stainless steel C-18 column (Nucleosil^®^100-5C18 (Suppleco series), Millipore Sigma, Burlington, MA, USA). The mobile phase was prepared using 0.2% TEA (pH 3.1) as the aqueous phase and acetonitrile (ACN) as the organic phase at a 20:80 ratio [35]. The pH of the aqueous component was adjusted to 3.1 using o-phosphoric acid before mixing it with ACN. The flow rate was set to 1 mL/min and 5 µL of the sample was injected for each run. The run time was set to 7 min considering the retention time of ibuprofen for this method (4.9 min). Detection of ibuprofen was performed using an ultraviolet-visible spectrophotometer (Agilent 1100 series, Agilent Technologies, Santa Clara, CA, USA) at a wavelength of 221 nm. Finally, the estimation of ibuprofen was performed using a calibration curve ranging from 1–64 µg/mL (R^2^ = 0.999).

### 2.8. Statistical Test and Dissolution Kinetics

Statistical assessment of the dissolution profiles was critical to show the impact of the process variable under investigation on the performance of the tablets. At first, the calculation of the similarity factor (*f_2_*) was considered to highlight the difference between the release kinetics of the tablets with different fills and polymers. Although *f_2_* is an effective tool to prove bioequivalence, it failed to depict the extent of dissimilarity in this case. Further, to investigate whether the difference between the dissolution profiles and hence the performance was significant, multivariate analysis of variance (MANOVA) analysis was considered and conducted using JMP^®^ software (JMP^®^ Pro 14, SAS Institute Inc., Cary, NC, USA). Briefly, a multivariate approach (MANOVA) was applied as it tested whether the difference amongst the percent dissolved at each time level was significant. First, without considering the different tablet characteristics (time), and then among the tablet characteristics (fill density and polymer used) with regards to the percent dissolved depending on time (time*tablet characteristics). This depicted whether the dissolution profiles of the different tablets were parallel [36]. The Wilks lambda statistic was preferred to obtain *p*-values. For the second step, a single group univariate repeated measures analysis of variance (univariate ANOVA) was applied. This time, the percent dissolved were tested separately at each time point to see if there were differences among the tested tablets (each group constituted of tablets having a certain grade of the polymer and a certain fill density) [36]. Finally, a Pearson’s correlation test was performed between two dependent variables (fill density and total % drug release) for tablets manufactured from the same polymeric filament and for tablets across all the different polymeric groups to confirm the broader impact of fill density on the release and to determine if there was any correlation between the fill density and the % drug release from the tablets. To evaluate the kinetics of drug release, profiles of all the tablets were fitted on curves of the mathematical drug release models (Zero-order kinetics, First-order kinetics, Higuchi model, Korsmeyer–Peppas, Hixson–Crowell, and Weibull function) and R^2^ values for each of the curves were obtained using an open source software KinetDS.

## 3. Results

### 3.1. Preparation of Drug-Loaded FDM Filaments via HME

Optimized HME processing was implemented to produce various filaments containing drug and polymeric carriers. Based on the thermal events observed during the screening process, the processing temperature for the manufacturing of the ASDs was set to 130 °C. After evaluating the compatibility of drug-polymer miscibility by applying theoretical structural orientation based prediction model of Hansen Solubility Parameters (∆δ = 4.11 MPa^0.5^ i.e., <7) [23] of ibuprofen (δ_t_ = 19.89 MPa^0.5^) [37] and HPMC-AS polymers (δ_t_ = 24 MPa^0.5^) [38], it was confirmed that the drug-polymer pairs were highly likely to be miscible and thus form a solid-dispersion. The theoretical evaluation of miscibility before manufacturing ASDs is crucial to predict the possibility of a crystalline drug to convert into its amorphous counterpart by balancing the energy required for intramolecular interaction within a drug to that of intermolecular interaction between that drug and a carrier matrix such as a polymer [23]. This also helps predict potential solubilization, and thus stabilization of a drug in ASDs during the HME process. The processing temperature was further confirmed from the mDSC of the produced ASDs. While manufacturing the filaments at 130 °C it was observed that the diameter increased at a higher RPM and feed rate which was due to the high viscosity of the polymer and the pressure build-up at the die, rendering them unsuitable for FDM printing. To maintain the diameter of the filament (2.85 ± 0.06 mm) and its applicability for 3D printing the RPM was reduced to 50, and the torque and die pressure were constantly monitored (within the range as mentioned in Table 1). Another alternative to solving this problem was increasing the zone temperatures, but this was avoided even though ibuprofen is not thermolabile. Post manufacturing, the drug content uniformity for all the filament batches was investigated and the potency was found to be within 98–102% of the expected drug content. Nonetheless, all formulations developed via optimized HME processing exhibited optimum properties and features for FDM 3D printing which was later assessed during the printing process.

### 3.2. Solid-State Characterization

**Figure 2:**
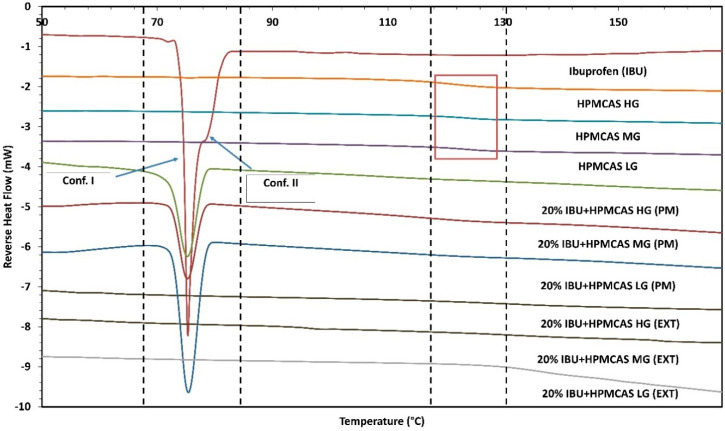
Modulated differential scanning calorimetry (mDSC) overlay. (PM = physical mixture, EXT = extruded filaments).

Thermal analysis of the pure drug exhibited a thermal transition beginning at 73 °C, i.e., the melting point of the drug. The glass transition temperature (*T*_g_) for HPMC-AS HG, MG, and LG was observed between 120 and 130 °C as a function of the polymeric compositions. As seen in Figure 2, the physical mixtures still depict the melting peak of the drug at 73 °C with slightly lower intensity whereas the extruded filaments do not exhibit any such thermal phenomenon, confirming the amorphous conversion of the drug moiety and the successful formation of ASDs.

**Figure 3:**
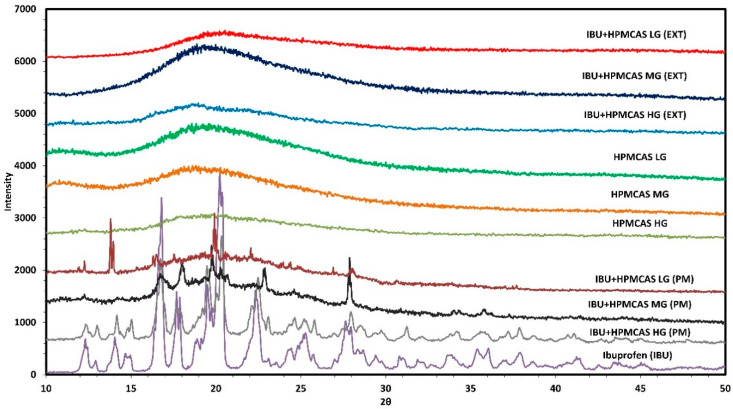
X-ray powder diffraction (pXRD) overlay. (PM = physical mixture, EXT = extruded filaments).

To further confirm the formation of the ASDs all the samples were analyzed using pXRD analysis. As can be seen in Figure 3, the drug is highly crystalline which is depicted by the distinct diffraction pattern and characteristic peaks at different 2θ positions. In contrast, all three different grades of HPMC polymer exhibited a halo shape due to its amorphous nature. It can also be observed in Figure 3 that all the significant 2θ peaks of ibuprofen (12.2°, 16.6°, 19.0°, and 22.3°) [39] are still visible in the physical mixture of the drug and polymer, whereas the peaks have disappeared in the extruded samples due to the amorphous conversion of the drug post-HME processing [40,41].

**Figure 4:**
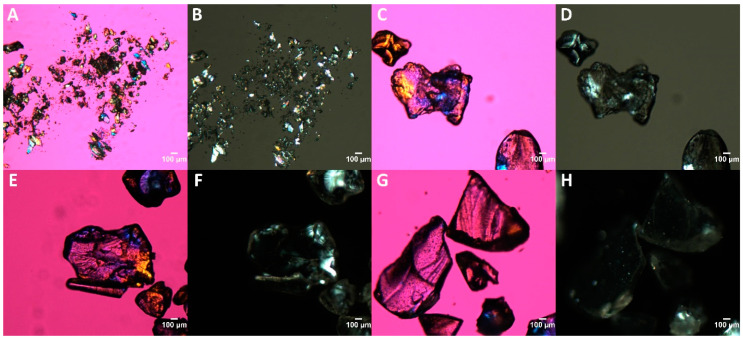
Polarized light microscopy (PLM) of: (**A**) pure crystalline ibuprofen (530 nm compensator), (**B**) pure crystalline ibuprofen (Dark background), (**C**) 20% ibuprofen + HPMC-AS HG extrudates (530 nm compensator), (**D**) 20% ibuprofen + HPMC-AS HG extrudates (Dark background), (**E**) 20% ibuprofen + HPMC-AS MG extrudates (530 nm compensator), (**F**) 20% ibuprofen + HPMC-AS MG extrudates (Dark background), (**G**) 20% ibuprofen + HPMC-AS LG extrudates (530 nm compensator), and (**H**) 20% ibuprofen + HPMC-AS LG extrudates (Dark background).

The distribution of the drug in the polymeric matrix and any traces of crystallinity in the extruded filaments were observed using PLM analysis [42]. From Figure 4, the bulk drug exhibits birefringence in both lights (Figure 4A) and dark conditions (Figure 4B) due to its crystalline nature and property to refract light. In contrast, even though the drug distribution in the extruded samples can be seen in the light conditions (Figure 4C,E,G), the drug has lost its property of birefringence when observed under dark conditions (Figure 4D,F,H), which is due to the lack of crystallinity. The observations made from the mDSC, pXRD, and PLM results assert the formation of ASDs. Any traces of birefringence observed in the ASDs under PLM are due to the semi-crystalline backbone of HPMC-AS as discussed by Davis A. and colleagues (in the press, 2020) [43].

**Figure 5:**
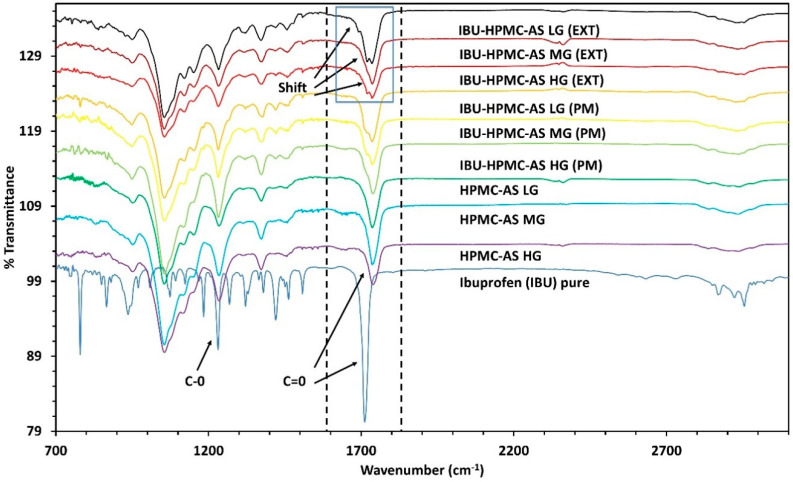
Fourier transform–infrared spectroscopic (FT–IR) spectrum overlay. (PM = physical mixtures, EXT = extruded filaments).

After confirming the formation of ASDs, the intramolecular interactions between the drug and the polymers in the extruded filaments were observed by conducting FT–IR analysis. The spectra obtained by FT–IR shed light on the mechanism of formation and stabilization of the ASDs. In Figure 5, the band present at 1710 cm^−1^ for the pure drug is due to the ‘C=O’ stretching present in the acid functional group [40,44]. The acid functional group in the drug has the most potential to interact with other molecules due to the partial negative charge on the oxygen atom. It also plays a crucial role in recrystallization [45]. The FT–IR spectrum shows a peak shift towards a higher wavenumber which may be due to the interaction of the acid functional group with the polymer, which may contribute towards stabilizing the ASDs [46].

### 3.3. Texture Analysis

**Figure 6:**
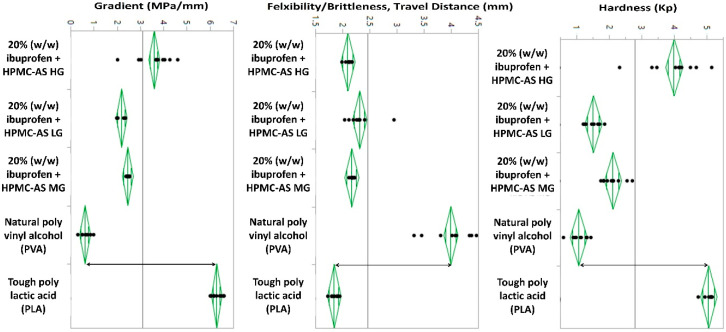
Graphical comparison textural parameters of the test and reference filaments.

After confirming the formation of ASDs, the next step was to check the compatibility of the extruded filaments to the FDM printing process, and the feasibility of printing the tablets. On conducting the texture analysis of the produced filaments, it was observed that HPMC-AS HG filaments had the highest hardness (Kp) and the lowest flexibility, whereas HPMC-AS LG observed the lowest hardness and the highest flexibility amongst the drug-loaded filaments (Table 3). HPMC-AS MG exhibited intermediate properties. The tough PLA filaments and the natural PVA filaments, due to their opposite nature provided a range for the acceptable numeric values of hardness and flexibility. This range can be observed in both Table 3 and Figure 6 where the two-sided arrows depict the values demonstrated for the two reference materials under discussion. It can be observed clearly in Figure 6 that all the extruded filaments fall within the range drawn by the two reference materials used.

The third parameter i.e., ‘gradient’ is defined as the amount of force per unit area i.e., the pressure applied times the distance traveled by the blade after contacting the filament’s surface. This parameter can be considered as the cumulative force required by the blade to travel 15 mm against the resistance provided by the filament. That being said, the tougher the filament, the higher the gradient. This correlation can be observed between the hardness of the filament and the attained value for the gradient.

### 3.4. Morphological Characterization

**Figure 7:**
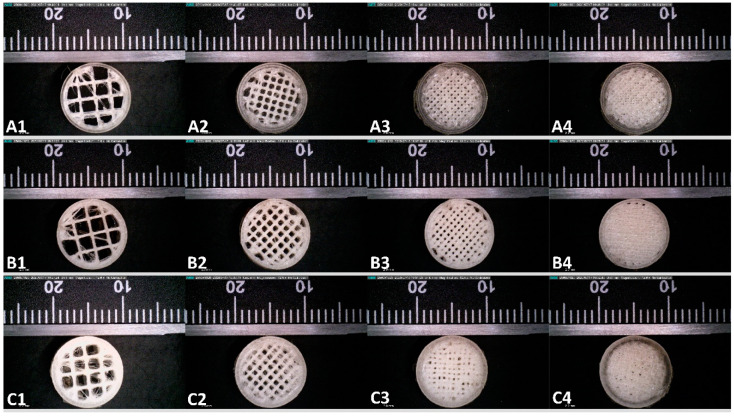
Digital microscopy of 20% *w*/*w* ibuprofen loaded (A) HPMC-AS HG 3-D printed tablets with 20% infill density (**A1**), 40% infill density (**A2**), 60% infill density (**A3**), and 80% infill density (**A4**). (B) HPMC-AS MG 3-D printed tablets with 20% infill density (**B1**), 40% infill density (**B2**), 60% infill density (**B3**), 80% infill density (**B4**). (C) HPMC-AS LG 3-D printed tablets with 20% infill density (**C1**), 40% infill density (**C2**), 60% infill density (**C3**), and 80% infill density (**C4**).

The tablets displayed in Figure 7 show the differences between infills with which the test tablets were printed. From the figure, it can be observed that 20% of infill has openings across the structure which would allow the buffer to interact with the core of the tablets during the dissolution studies. Whereas, the tablets with 80% infill have no gaps for the media to interact with the core which should lead to a more sustained release. The dimensions of the tablets were also measured while conducting the morphological evaluation and it was observed that the tablets had uniform dimensions.

### 3.5. In Vitro Drug Release Testing

**Figure 8:**
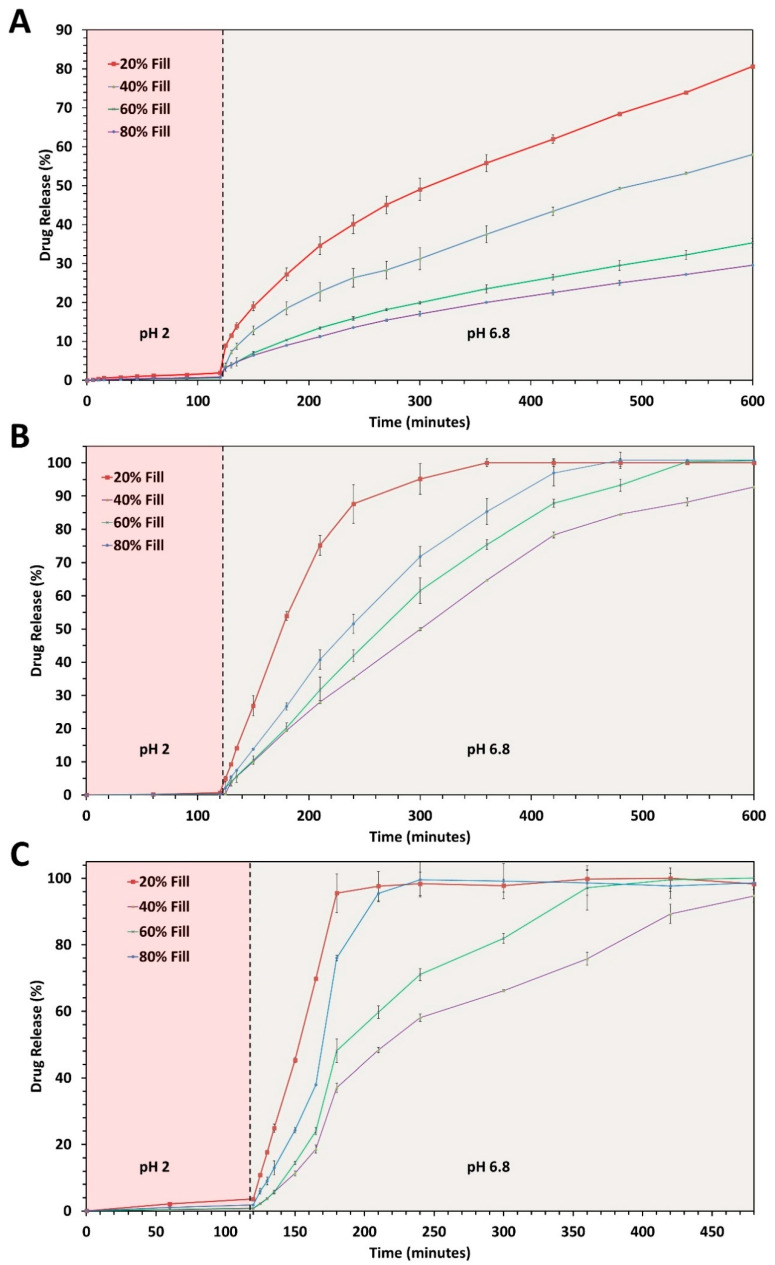
(**A**) The in vitro release profiles of 20% *w*/*w* ibuprofen loaded HPMC-AS HG 3-D printed tablets with different infill densities. (**B**) The in vitro release profiles of 20% *w*/*w* ibuprofen loaded HPMC-AS MG 3-D printed tablets with different infill densities. (**C**) The in vitro release profiles of 20% *w*/*w* ibuprofen loaded HPMC-AS LG 3-D printed tablets with different infill densities.

The pH shift dissolution testing protocol was developed due to the pH-dependent solubility of HPMC-AS polymers. As discussed before, HPMC-AS HG has the highest number of acetyl substitutions and the lowest number of succinoyl substitutions. Due to the chemistry of HPMC-AS HG, its pH threshold for solubilization (pH > 6.8) is the highest amongst all the HPMC-AS polymers and hence is usually considered for colon targeted dosage forms. For ASDs the drug release is controlled by either the hydration rate of the polymer (in case of swellable systems) or the solubilization. Due to the high pH threshold, it was observed that the HPMC-AS HG tablets were forming a viscous gel layer enabling drug release via diffusion, instead of dissolving in the buffer and releasing the drug by erosion. The formation of this gel layer was based on the rate of hydration, which was dependent on the buffer-accessible surface area of the tablet. The 20% HPMC-AS HG tablets, due to their low fill density, allowed the buffer to hydrate the core of the tablets, leading to quicker hydration and thereby faster as well as more complete drug release. In contrast, tablets of HPMC-AS HG with an 80% infill depicted a slower and more controlled drug release. This is evident in Figure 8A and it can also be seen that the % drug release over time reduces with the increasing fill density. Statistical proof of this correlation has been established in the later parts of this manuscript.

Moving forward, the HPMC-AS MG tablets have an intermediate amount of acetyl and succinoyl substitutions and hence a lower pH threshold (pH > 6) for solubilization as compared to HPMC-AS HG. HPMC-AS MG based tablets observed complete solubilization and hence no gel layer formation was observed. The complete solubilization of the tablets is also apparent by the complete release of the drug from the tablets. It is interesting to observe a drug release of more than 85% (~32 mg) in less than 2 h of pH shift for the 20% infill tablets, whereas 40% infill demonstrated a drug release of >85% only after 3 h of pH shift (Figure 8B). Moreover, the trend observed for HPMC-AS HG is still prominent in HPMC-AS MG where tablets with 20% infill released the drug the fastest and those with 80% infill observed a more sustained release as compared to the 20%, 40%, and 60% infill tablets. The pH threshold for HPMC-AS LG polymers is the lowest i.e., pH > 5.5, thereby the HPMC-AS LG tablets with 20% infill observed the fastest drug release (<1 h after pH shift) when compared with other infills of the same polymer and tablet performances of the other polymers (Figure 8C). Even though HPMC-AS LG has the lowest pH threshold it still follows the same trend observed by the other two polymers where the release of the drug from the tablets with lower infill is faster as compared to the release from tablets with higher infills. These findings are in line with the initial hypothesis of this research project that the fill density of the tablets have a major impact on the drug release behavior and thereby the overall performance of the 3-D printed tablets, irrespective of the mechanism of release i.e., diffusion or solubilization.

In terms of the drug load (Table S2), for HPMC-AS HG, even though the tablets with the lowest infill had the least amount of the drug (35.9 ± 0.23 mg) and the tablets with the highest infill had the most (66.86 ± 0.13 mg), tablets with the 20% infill released >80% of the drug, i.e., ~28 mg of the drug, whereas, tablets with an 80% infill release only 25% of the drug, i.e., ~16 mg. Similar trends were observed for HPMC-AS MG, where the 20% infill (37.15 ± 0.62 mg) released >85% (~32 mg) of the drug in less than 4 h, in contrast, the tablets with an 80% infill (69.6 ± 1.62 mg) released only about 30% (~21 mg) of the drug in that time. Although HPMC-AS LG observed a faster release as compared to the other polymers for all the infills the trend was still prominent where 100% (27.50 ± 1.33 mg) of the drug was released from the tablets with 20% infill in less than an hour after the pH shift, but only 35% (~22 mg) of the drug was released for the tablets with an 80% infill by that time point. These differences might appear small for a drug like ibuprofen but are massive for drug classes such as antihypertensives (atorvastatin, nifedipine), or anti-cancer (paclitaxel), where the difference of merely 10 mg changes the dosage form from an immediate release to a sustained/controlled release dosage form.

**Figure 9:**
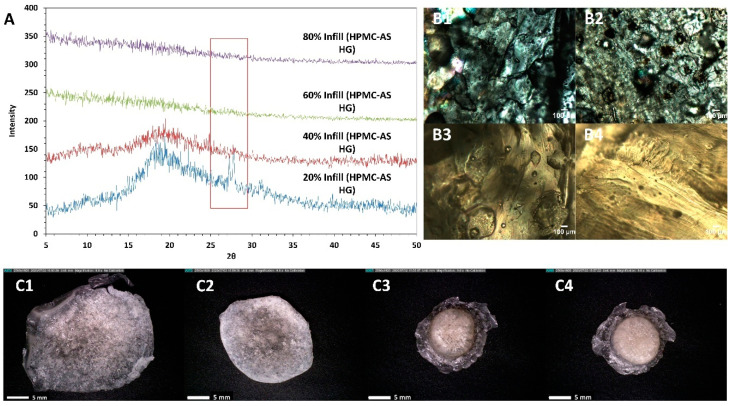
(**A**) pXRD of 20% *w*/*w* ibuprofen loaded HPMC-AS HG 3-D printed tablets collected after dissolution testing. (**B**) PLM of 20% *w*/*w* ibuprofen loaded HPMC-AS HG 3-D printed tablet post-dissolution testing with 20% infill density (**B1**), 40% infill density (**B2**), 60% infill density (**B3**), and 80% infill density (**B4**). (**C**) Digital microscopic images of 20% *w*/*w* ibuprofen loaded HPMC-AS HG 3-D printed tablet post-dissolution testing with 20% infill density (**C1**), 40% infill density (**C2**), 60% infill density (**C3**), and 80% infill density (**C4**).

One probable reason other than the impact of fill density to justify the stunted release of some of the tablets was the formation of a crystalline drug rich layer surrounding the tablet, thus inhibiting the release of the amorphous drug from the core [47]. To investigate whether this was the reason for the observed reduction in drug release for HPMC-AS HG (tablets prepared with HPMC AS-MG, and HPMC AS-LG disintegrated completely) the tablets post-dissolution testing were collected and analyzed for birefringence and X-ray diffraction. On analysis, no traces of crystallinity were found on the isolated tablets as seen in Figure 9. Although the 20% infill tablet residue displayed a peak at a 2θ of 27.3°, the intensity of this peak was negligible. Moreover, the drug in the more concerning samples such as with a higher amount deposited (40% infill, 60% infill, and 80% infill), was found to have retained its amorphous form post-dissolution. The peak observed in the 20% samples could have been due to the recrystallization of the dissolved drug in close vicinity of the surface of the tablet when it was being isolated for analysis. Nevertheless, all other formulations retained the amorphous nature of the crystalline drug. Another probability for the hindered release could be the weak intermolecular interactions between the polymer and the drug observed on the FT–IR analysis, although this is highly unlikely. HPMC-AS is prone to have inter-molecular interactions with ibuprofen’s carboxylic acid functional group which would be strong enough to stabilize it and hence prevent recrystallization on storage, but not strong enough to hinder the release of the drug. As observed in a study by Ewing and colleagues (2014) with indomethacin and HPMC, it was capable to prevent recrystallization of indomethacin on storage and did not hinder the release of indomethacin during the in vitro testing as its interaction with the dissolution media were considered to be stronger as compared to the weak interactions it has with the drug, thereby releasing the drug in the medium [48]. Although there have been cases reported with other drug-polymer pairs such as polyethylene glycol and indomethacin, where the drug recrystallizes during the storage and release testing, which is attributed to the solubilization capacity of the polymer and the compatibility of the drug and the polymer [23]. In our case, as per the theoretical parameters discussed previously the drug and the polymer are compatible with one another, which is also supported by the solid-state characterizations conducted during the study. Moreover, from the release studies, it can be seen that the polymer can maintain the supersaturation of the drug throughout the test.

### 3.6. Statistical Test and Dissolution Kinetics

**Figure 10:**
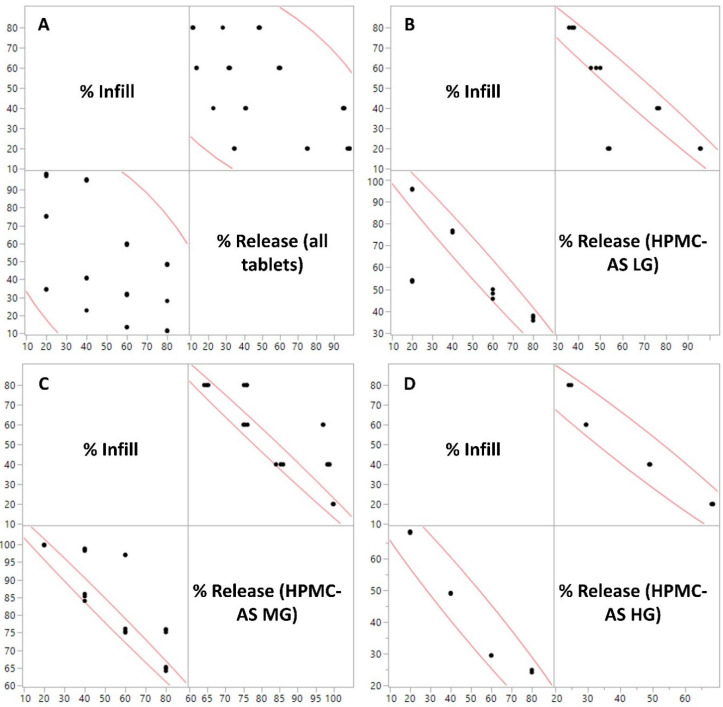
Pearson’s correlation between % Infill of the 3D printed tablets and % Release from the tablets for (**A**) All polymer-infill combinations, (**B**) HPMC-AS LG, (**C**) HPMC-AS MG, and (**D**) HPMC-AS HG).

The MANOVA analysis was conducted for the percentage (%) drug release at all the time points taken (125–480 min) where the said data was considered as a dependent variable and the formulation composition, i.e., the combination of the polymer and % fill were considered as the independent variable. The interaction between the said variables was found to be significant both between groups (F(11, 24) = 15,271.147, *p* < 0.0001) and within groups (Wilks’ Lambda = 8.948 × 10^−21^, F (110, 125.6) = 544.1856, *p* < 0.0001), i.e., the independent variable (the grade of polymer used and the % fill of the 3-D printed tablets) had a significant impact on the % drug release from the tablets over time. To see if the drug release at each time point was significantly different amongst the samples, ANOVA analysis was conducted for each time point. It was observed that there was a significant difference between the drug release at each time point (Table 4). Further, a Pearson’s correlation test was performed between the variables (cumulative % drug release and % fill) to see if there was any correlation between the two variables. The test was conducted for tablets made of the same polymer (to isolate the effect of fill density) but having different infills (Figure 10B–D), and across all the polymers where the tablets with the same infill were pooled in one group (Figure 10A). There was a strong correlation between the individual polymers and the infill (r > −0.99; *p* < 0.0001) which further bolsters the hypothesis, i.e., not only fill density has a significant impact on the performance of the tablet but also the impact has an observable trend.

There was a moderate correlation when all the polymers were considered (r > −0.5440; *p* = 0.0006). The probable cause for the reduction in the ‘r’ value is the addition of another variable i.e., the type of polymer which also has an impact on the release performance of the tablets. It should also be noted that the correlation coefficient is negative, i.e., the increased infill density leads to a reduction in the cumulative drug release from the 3-D printed tablets.

The dissolution profiles of all the tablets were curve-fitted to mathematical drug release models (Table 5). The HPMC-AS HG release profiles fit well with the Higuchi model, whereas there is a declining trend of correlation to the Higuchi model observed in HPMC-AS MG and HPMC-AS LG which is understandable since the Higuchi model considers dissolution, as well as shape change of the system insignificant and, is more focused on the release via diffusion [49].

This mechanism holds for the HPMC-AS HG polymer since there is the minimal dissolution of the tablets observed and the key mechanism of release is believed to be diffusion as the shape of the system does not change [50]. It can be seen that all the profiles fit well with the zero-order release kinetics (Table 5). This is because the drug release is not due to the immediate disaggregation of the dosage form as seen in immediate release or orally disintegrating tablets, but rather due to the slow release of the drug [49]. This slow-release can be attributed to the sink conditions maintained throughout the study and the inherent properties of ASDs where the drug releases occur by slow surface dissolution of the tablets. All the drug release profiles also fit the Korsmeyer–Peppas model. This model is used to describe the drug release from polymeric systems such as hydrogels and considers both Fickian and non-Fickian drug release mechanisms which also explains why the profiles fit the zero-order release kinetics [51]. Since in this case, the solubility of the tablets is largely dependent on the pH threshold of the participating polymers, the tablets observe a combination of Fickian and non-Fickian release which is taking into consideration by the Korsmeyer–Peppas model. The model also takes into account the change in geometry as well as the shape over time and hence, can explain the release behavior of HPMC-AS MG and HPMC-AS LG where the assumptions set by the Higuchi equation are not followed [51].

## 4. Discussion

Few studies in the past have focused on the print parameters and their impact on the performance of the tablets since most of the studies are focused on the formulation development and filament composition aspect for the pharmaceutical 3-D printing research. The presented study provides in-depth insights supported by statistical and mathematical models in regard to the impact of fill density on the performance of 3-D printed dosage forms. This information can be used to design dosage forms without changing the filament composition to release the drug as per the patient’s requirements, thereby finding application in the field of personalized medicine. Technologies such as shell-core for the enteric coating of acid-labile drugs [19], hollow systems or multi-compartment systems [30], designs with abuse-deterrent properties [52] and platforms testing different polymers such as thermoplastic polyurethanes with high drug loads of theophylline and metformin [53], polyvinyl alcohol polymers (PVA) [29], polyvinyl pyrrolidone (PVP) [40,54], methacrylate polymers [25], polyethylene oxide polymers, and HPMC based polymers [12,22] have been extensively studied for their suitability to 3-D printing platforms. In the study by Goyanes, A. and colleagues (2015)*,* the main focus was to investigate the feasibility of using FDM processing in the development of modified-release formulations of two aminosalicylate isomers used in the treatment of inflammatory bowel disease (IBD) i.e., 5-aminosalicylic acid (5-ASA, mesalazine) and 4-aminosalicylic acid (4-ASA). The study was successful in controlling the release of the drugs by using a commercially manufactured PVA filament, where the drug was absorbed on to the said filament from an ethanolic drug solution. On testing, the performance of the FDM printed tablets at different fill densities (10%, 50%, and 90%) as a secondary objective of the study it was observed that the print parameter had some role in controlling the release of the drug. The study concluded that the 10% infill tablets observed a complete release after 4 h dissolution, but both the 50% and 90% infill tablets showed burst release followed by slow release [55]. Although this study provides some insights on the impact of fill density on release, it cannot explain the phenomenon for matrix-based systems or amorphous solid dispersions as the method of preparation of the filaments is fundamentally different, and the drug is absorbed superficially, which gives us reason to believe that the drug release was dominantly dependent on the drug’s intrinsic properties of diffusion or solubilization.

In contrast, the present study was focused on the ASDs of a BCS class II drug which was prepared using HPMC-AS based polymers, where the drug was molecularly dispersed in the polymeric matrix, and the rate-limiting factor for the release of the drug was the solubility/rate of hydration of the polymer, which was dependent on the fill density of the tablets and not the intrinsic properties of the drug. Thereby, this study can be used to explain the release behavior of any drug dispersed in such a hydrophilic polymeric matrix concerning the fill density set during the slicing of the devices/tablets. In retrospect, Verstraete G., and colleagues (2018) have conducted a similar study where high drug-loaded (>30%, *w*/*w*), thermoplastic polyurethane (TPU)-based dosage forms were tested for their sustained release properties using FDM 3-D printing. As a part of this study Verstraete, G. and colleagues prepared tablets with different fill densities (25%, 50%, 75%, and 100%) and compared them. They drew observation-based conclusions where the release profiles were justified by the type of polymer used, the drug type, and the porosity of tablets [53]. Yang and colleagues (2018) and Goyanes and colleagues (2017) also conducted studies that have used ethyl cellulose and HPMC-AS based matrices, respectively [12,56]. These publications have uncovered some association between infill and release, but it is crucial to determine the significance and correlation statistically of this association to further justify the release behavior FDM 3-D printed devices/dosage forms.

The present study isolated one type of drug (weakly acidic and poorly water-soluble) and one class of polymer i.e., HPMC-AS polymers, and tested the impact of fill density on all the available grades (HG, MG, and LG) of the said polymer on the release of the drug. The study found that the polymers successfully stabilized the ASD through weak intermolecular interactions and that the surface recrystallization of the drug in the dissolution vessel was not responsible for the release behavior of the drug in this case. As described by Que and colleagues (2019) and Alonzo and colleagues (2010) this phenomenon is commonly observed in ASDs even more so with higher drug loads, therefore there is a chance that on increasing the drug loading in the filaments, this phenomenon would be more apparent [47,57]. An increase in the drug load might also test the polymer’s capabilities of maintaining the supersaturation of the drug in the release medium, although at the current drug load the polymer was able to stabilize the drug in the solution for over 8 h. From the observations, it seems that the major mechanism dictating this release behavior is the rate of hydration of the HPMC-AS polymers which is dependent on the accessible surface area [39]. Supporting this claim, the study found a significant impact and correlation between the fill density and drug release from the polymer whilst eliminating any other factor impacting or contributing to the drug release. The results provide a deeper insight into the impact of the process parameters and process variables on the performance of the 3-D printed tablets. The study also shows that the performance and dynamics of the drug release can be changed without changing any of the formulation parameters (drug load, polymer, and composition). Furthermore, these findings can be translated into applications for personalized therapy where the same filaments with a fixed drug load can be used to make dosage forms with different release behaviors as per the patient’s needs and therapeutic requirements. This study can be used as a precedent for any further studies investigating the application of HPMC-AS polymers in FDM based 3-D printing. Although the present study provides a brief insight into the possible mechanism for explaining the impact of fill density on the release behavior of the tablets, further studies and advanced characterizations are required to pinpoint the exact mechanism. This will in turn provide better control over the discussed concept. It will also help in designing dosage forms by varying print parameters to get the desired release profile. Moreover, the study covers one class of thermoplastic polymers and its grades as well as one drug type. Further studies with different types of drugs (weakly basic-poorly soluble/weakly acidic-water soluble/weakly basic-water soluble) and other FDM compatible thermoplastic polymers would provide a complete understanding of the extent to which the discussed process variable impacts the performance of 3-D printed dosage forms.

## 5. Conclusions

This study demonstrated the use of fill density as an effective tool for controlling the drug release from 3-D printed devices. The ASDs containing 20% ibuprofen in HPMC-AS polymers were successfully manufactured and found suitable for FDM-based 3-D printing applications. Moreover, the solid-state characterization of the filaments asserted the amorphous conversion of the drug and aligned with one another providing a deeper insight into the mechanism of stabilization of the ASDs under discussion. Tablets with different infills were successfully prepared and their morphology was assessed before exposing them to in vitro dissolution testing. These tablets observe a combination of Fickian and non-Fickian release which is taken into consideration by the Korsmeyer–Peppas model. The model also takes into account the change in geometry as well as the shape over time and hence, can explain the release behavior of the drug from the HPMC-AS polymers. The release studies provided a richer understanding of the impact of % infill on the performance of the tablets and the significance of these observations was further reinforced by statistical evaluations and kinetic modeling, thereby disclosing the strong negative correlation between the fill density and drug release. These findings enhance the scope of FDM 3-D printing for producing on-demand patient-specific dosage forms and takes the field one step closer to achieving the terminal goal of personalized therapy.

## Figures and Tables

**Table 1:** Hot-melt extrusion (Nano-16, Leistritz) processing parameters.

Parameters	Values
Zones	4
Temperature (°C)	130 °C throughout
Screw RPM	50
* Feed rate (RPM)	500 ± 50 (5 g/min)
Observed torque on equilibration	5.30 ± 0.27 N·m
Residence time (min)	2.3 ± 0.2
Die pressure	55 ± 10 psi

* The feeder should be calibrated with the material in use before every extrusion as it may change depending on the make and model of the feeder and the properties of the fed material.

**Table 2:** Print parameters for the drug-loaded 3-D printed tablets.

Print Parameters	Values Set
Print temperature	175 °C–180 °C
Bed temperature	60 °C
Print speed	70 mm/s
Layer height	0.1 mm
Wall thickness	2 mm
Number of walls	2
Fan speed	70%
Infill pattern	Lines
Top/bottom layers	None
Print core	AA 0.4

**Table 3:** Descriptive statistics for the attained textural properties of the tested drug-loaded filaments and reference materials.

Result Parameters	Hardness (Kp)	Brittleness/Flexibility, Travel Distance (mm)	Gradient (MPa/mm)
20% (*w*/*w*) ibuprofen + HPMC-AS HG			
Average	3.99	2.09	3.582
Standard Deviation (S.D.)	0.76	0.06	0.715
Coefficient of Variance (C.V.)	18.94	3.1	19.963
20% (*w*/*w*) ibuprofen + HPMC-AS MG			
Average	2.11	2.16	2.445
Standard Deviation (S.D.)	0.30	0.03	0.057
Coefficient of Variance (C.V.)	14.3	1.55	2.339
20% (*w*/*w*) ibuprofen + HPMC-AS LG			
Average	1.51	2.31	2.179
Standard Deviation (S.D.)	0.21	0.23	0.153
Coefficient of Variance (C.V.)	13.91	10.05	7.02
Tough PLA filaments			
Average	5.05	1.84	6.269
Standard Deviation (S.D.)	0.13	0.07	0.204
Coefficient of Variance (C.V.)	2.65	3.55	3.258
Natural PVA filaments			
Average	1.05	3.99	0.607
Standard Deviation (S.D.)	0.24	0.36	0.189
Coefficient of Variance (C.V.)	22.44	9.02	31.1

**Table 4:** Univariate ANOVA results for each time point tested separately against the tablet composition.

Time Point	Result
125	F (11, 24) = 1016.780, *p* < 0.0001
130	F (11, 24) = 21,291.52, *p* < 0.0001
135	F (11, 24) = 3926.784, *p* < 0.0001
150	F (11, 24) = 28,952.28, *p* < 0.0001
180	F (11, 24) = 376.868, *p* < 0.0001
210	F (11, 24) = 25,253.55, *p* < 0.0001
240	F (11, 24) = 20,503.98, *p* < 0.0001
300	F (11, 24) = 13,697.65, *p* < 0.0001
360	F (11, 24) = 13,738.84, *p* < 0.0001
420	F (11, 24) = 11,424.00, *p* < 0.0001
480	F (11, 24) = 15,084.65, *p* < 0.0001

**Table 5:** Curve-fitting of release profiles on mathematical drug release models.

Formulation Compositions (20% *w*/*w* Ibuprofen)	Kinetic Models (R^2^)						
	% Infill	Zero-Order Kinetics	First-Order Kinetics	Higuchi Model	Korsmeyer–Peppas	Hixson–Crowell	Weibull Function
HPMC-AS HG	20	0.9522	0.7795	0.9992	0.9982	0.8497	0.9859
	40	0.9704	0.7604	0.9784	0.9955	0.8546	0.9934
	60	0.9679	0.7915	0.9765	0.9947	0.8661	0.9926
	80	0.9703	0.815	0.9928	0.9928	0.8809	0.9899
HPMC-AS MG	20	0.8587	0.6807	0.7353	0.9758	0.7549	0.9136
	40	0.959	0.7183	0.6195	0.9904	0.8347	0.9004
	60	0.9683	0.7487	0.5841	0.9964	0.8558	0.8915
	80	0.9759	0.6322	0.4707	0.9404	0.828	0.9679
HPMC-AS LG	20	0.9978	0.9456	0.6613	0.9942	0.9812	0.9415
	40	0.935	0.8492	0.514	0.9754	0.8973	0.9352
	60	0.8884	0.6397	0.5128	0.9634	0.7425	0.9869
	80	0.9171	0.6556	0.5979	0.9706	0.7648	0.9885

## Supplementary Materials

The following are available online at https://www.mdpi.com/2073-4360/12/9/1872/s1, Table S1. Recipe for preparing buffers for pH shift dissolution studies. Table S2. Tablet weights used for the dissolution studies.
