# Supplementary material for: Novel On-Demand 3-Dimensional (3-D) Printed Tablets Using Fill Density as an Effective Release-Controlling Tool
Source: Polymers (Basel). 2020 Aug 20;12(9):1872. doi: 10.3390/polym12091872 (PMC7564432; doi:10.3390/polym12091872)
Supplement: Supplementary file 1 [file polymers-12-01872-s001.pdf]

Supplementary data

Table S1. Recipe for preparing buffers for pH shift dissolution studies.

Components	Molecular formulae	Mol. Wt. (g/mol)	molarity (M)	Amount	Final volume
**Hydrochloric acid (HCl)-Potassium Chloride (KCl) buffer (*pH 2, 0.1M)**					
**hydrochloric acid (1N)**	HCl	36.46	0.02	15 mL	750 mL
**potassium chloride**	KCl	74.55	0.10	5.591 g	
**Phosphate buffer (*pH 6.5, 0.1M)**					
**sodium phosphate dibasic**	Na_2HPO_4	141.96	0.049	6.260 g	150 mL
**sodium phosphate monobasic monohydrate**	$NaH_2PO_4.H_2O$	137.99	0.051	6.334 g	
**Total Volume (final)**					900 mL

*Adjust final pH using hydrochloric acid (HCl) or sodium hydroxide (NaOH)

Table S2. Tablet weights used for the dissolution studies.

Formulation composition	Tablet weight (mg)	Amount of drug (as per the drug content)
**20% ibuprofen-HPMC-AS HG (20% infill)**	176.4	35.28
	182.3	36.46
	179.8	35.96
**20% ibuprofen-HPMC-AS HG (40% infill)**	228.7	45.74
	226.4	45.28
	232.6	46.52
**20% ibuprofen-HPMC-AS HG (60% infill)**	286.9	57.38
	285.4	57.08
	277.6	55.52
**20% ibuprofen-HPMC-AS HG (80% infill)**	334.2	66.84
	336.6	67.32
	332.1	66.42
**20% ibuprofen-HPMC-AS MG (20% infill)**	185.6	37.12
	182.7	36.54
	188.9	37.78
**20% ibuprofen-HPMC-AS MG (40% infill)**	235.0	47
	233.1	46.62
	235.6	47.12
**20% ibuprofen-HPMC-AS MG (60% infill)**	291.7	58.34
	288.8	57.76
	293.4	58.68
**20% ibuprofen-HPMC-AS MG (80% infill)**	339.1	67.82
	350.6	70.12
	354.7	70.94
**20% ibuprofen-HPMC-AS LG (20% infill)**	136.2	27.24
	144.7	28.94
	131.6	26.32
**20% ibuprofen-HPMC-AS LG (40% infill)**	212.5	42.5
	212.7	42.54
	215.4	43.08

**20% ibuprofen-HPMC-AS LG (60% infill)**	277.3	55.46
	285.1	57.02
	288.9	57.78
**20% ibuprofen-HPMC-AS LG (80% infill)**	330.7	66.14
	325.4	65.08
	325.2	65.04
